# Age and Ethnic Differences in Volumetric Breast Density in New Zealand Women: A Cross-Sectional Study

**DOI:** 10.1371/journal.pone.0070217

**Published:** 2013-07-31

**Authors:** Lis Ellison-Loschmann, Fiona McKenzie, Ralph Highnam, Andrew Cave, Jenny Walker, Mona Jeffreys

**Affiliations:** 1 Centre for Public Health Research, Massey University, Wellington, New Zealand; 2 International Agency for Research on Cancer, Lyon, France; 3 Matakina Technology Ltd, Wellington, New Zealand; 4 Waitemata District Health Board, Auckland, New Zealand; 5 School of Social and Community Medicine, University of Bristol, Bristol, United Kingdom; Dartmouth, United States of America

## Abstract

Breast cancer incidence differs by ethnicity in New Zealand (NZ) with Māori (the indigenous people) women having the highest rates followed by Pakeha (people primarily of British/European descent), Pacific and Asian women, who experience the lowest rates. The reasons for these differences are unclear. Breast density, an important risk factor for breast cancer, has not previously been studied here. We used an automated system, Volpara™, to measure breast density volume from the medio-lateral oblique view of digital mammograms, by age (≤50 years and >50 years) and ethnicity (Pakeha/Māori/Pacific/Asian) using routine data from the national screening programme: age; x-ray system and mammography details for 3,091 Pakeha, 716 Māori, 170 Pacific and 662 Asian (total n = 4,239) women. Linear regression of the natural logarithm of absolute and percent density values was used, back-transformed and expressed as the ratio of the geometric means. Covariates were age, x-ray system and, for absolute density, the natural log of the volume of non-dense tissue (a proxy for body mass index). Median age for Pakeha women was 55 years; Māori 53 years; and Pacific and Asian women, 52 years. Compared to Pakeha women (reference), Māori had higher absolute volumetric density (1.09; 95% confidence interval [95% CI] 1.03–1.15) which remained following adjustment (1.06; 95% CI 1.01–1.12) and was stronger for older compared to younger Māori women. Asian women had the greatest risk of high percentage breast density (1.35; 95% CI 1.27–1.43) while Pacific women in both the ≤50 and >50 year age groups (0.78; 95% CI 0.66–0.92 and 0.81; 95% CI 0.71–0.93 respectively) had the lowest percentage breast density compared to Pakeha. As well as expected age differences, we found differential patterns of breast density by ethnicity consistent with ethnic differences seen in breast cancer risk. Breast density may be a contributing factor to NZ’s well-known, but poorly explained, inequalities in breast cancer incidence.

## Introduction

Breast cancer remains the most important cancer for New Zealand women, who experience high breast cancer incidence and mortality rates compared to many other developed countries [1] with breast cancer accounting for fully 28% of all cancer registrations and 16% of all cancer deaths among women in 2009 [2]. The burden of breast cancer is not shared equally across ethnic groups however [2]–[4] and while earlier data reported rates to be reasonably similar between Māori and non-Māori women [5]–[7], more recent work highlights an increasing incidence of breast cancer among Māori [2]–[3]. Time trend data for a 25 year period from 1981–2004 show that the incidence rate ratio of breast cancer in Māori increased from 7% higher than Pakeha to 24% higher (age-standardised rate ratio [SRR] 1.07 in 1981–86 and SRR 1.24 in 2001–04) [3]. During the same period, Pacific and Asian women had consistently lower incidence rate ratios (12% and 28% respectively) than Pakeha women with no marked changes in the differences between Pacific and Asian and Pakeha women seen over the time period studied.

Breast density, a measure of the relative amounts of dense and fatty breast tissue, is an important risk factor for breast cancer; women with dense breasts have been found to be four to six times more likely to develop breast cancer than those with fatty breasts [8]–[9]. Breast density is influenced by a range of factors including age [10], and menopausal status [11]. Variation in breast density has also been reported by ethnicity [12]–[15] but the reasons for this variation are not clear. In the United Kingdom (UK), density was found to be lowest in British South Asian women, intermediate in African-Caribbean women, and highest in white women [12]. United States (US) data show that, compared to white American women, African-American [13] and native Hawaiian [14] women have lower percentage breast density, which is partially accounted for by higher body mass index (BMI), while results for Asian women are inconsistent [13]–[15].

Quantitative methods to measure the area of the mammogram that is dense include visual assessment and computer-assisted thresholding methods [16]. In the clinical setting, the method most often used by radiologists is the American College of Radiology Breast Imaging Reporting and Data System (BI-RADS) [17]. More recent technology has moved to fully automated volumetric measures of breast density [18] which can be utilised with digital mammography only.

A national breast screening service has been offered in New Zealand since 1999. Currently, digital mammography is installed in four of the eight centres of the service, Breast Screen Aotearoa (BSA) [19]. The eligible age range for the screening programme is 45–69 years of age and two-yearly screens are recommended [19]. One fully automated system to measure breast density, Volpara™, is available as a research tool for use by BSA. Volpara™ provides an assessment of the percentage of dense tissue contained within the breast and a mapping of that percentage to a Volpara Density Grade (VDG) [20] which corresponds to the BI-RADS breast density categories, 1 to 4 [21]. The overall agreement between VDG and a radiologist's assessment of BI-RADS breast density category was approximately 70% [21].

No studies of breast density have previously been conducted in New Zealand, thus, there is an opportunity to obtain some baseline information about this important risk factor within the context of an ethnically diverse population. Since mammographic density distributions both contribute to breast cancer risk profiles [22] and affect likelihood of detection [23], breast density consequently may be an important determinant of ethnic inequalities in breast cancer incidence and survival. Additionally, given the age range for free breast screening, New Zealand is able to investigate breast density in both pre- and post-menopausal women. This paper reports the results of a pilot study to obtain novel data on the distribution of breast density in New Zealand women, by ethnicity and age, using Volpara™ on digital mammographic images already collected by one of the BSA Lead Providers, Breast Screen Waitemata Northland (BSWN).

## Material and Methods

### Ethics Statement

The study was approved by the Northern X Regional Ethics Committee (ref: NTX/11/EXP/112) of New Zealand. All analyses were carried out on routinely collected data, made available to the researchers in a de-identified form, which had already been obtained at the time of the mammogram by BSWN. The Northern X Regional Ethics Committee therefore accepted that written consent was not required by individual participants and granted approval for the study.

### Study Population

The aim of the study was to describe the distribution of breast density in a population of New Zealand women. New Zealand has a population of approximately 4.4 million [24] and comprises four main ethnic groups: Māori (15%), people from the Pacific islands (7%), Asian peoples (10%) and Pakeha (77%) [25]. Ethnicity was determined based on BSA protocol where women self select their primary ethnic group, hence, where more than one ethnic group was chosen, we have used the category selected by women as their primary ethnic group in these analyses. Thus, for the current study, we examined breast density according to ethnicity (Pakeha/Māori/Pacific/Asian) and age group (≤50 years/>50 years).

The participants were all women who underwent routine mammography screening during the period 3/12/2010–30/6/2011 at the Waitakere and Takapuna fixed sites and the mobile units operating in Northland as part of the BSWN programme. BSWN has an eligible screening population of approximately 100,000 women and screens around 35,000 women per year [26]. A unique identifier (the National Health Index [NHI] number) which is used within the New Zealand health system across a range of health services [27], was linked to the individual woman’s data from the BSWN programme. This linked information was known only to the Clinical Director of BSWN who is a Co-investigator on the study (Walker). Once all data were assembled, the NHI number was removed, and the dataset was made available to the study team in this de-identified form.

### Data Collection

Only data routinely collected by the BSA programme was available for the study which included: age at examination; self-identifed ethnicity; x-ray system (GE or Siemens) and mammography screen details using the medio-lateral oblique (MLO) view (left breast & right breast). The volume of the mammogram that appeared dense was estimated using the Volpara™ system version 1,4,1. This is a computer algorithm which models the x-ray physics in order to produce estimates of the total breast volume, the absolute dense volume, the volume of non-dense tissue and the percentage of the total breast volume that is dense (density percentage). Volpara™ works by using the physics parameters to estimate the x-ray attenuation between any pixel and the x-ray source. From that attenuation, it is possible to estimate the amounts of fat and fibroglandular (dense) tissue present [21].

Both GE and Siemens x-ray systems are in use in BSWN. Initial work indicated that the two x-ray systems gave slightly different results of Volpara™ density for the same women. Thus, a standardisation procedure was used for the Siemens data, using results from Volpara™ system version 1,4,2, looking at how the results changed between versions 1,4,1 and 1,4,2 and applying a correction factor to the absolute dense volume and the total breast volume estimates. Breast thickness was determined from the distance between the compression plates during mammography.

### Statistical Analysis

Absolute dense volume was divided into quintiles for analysis. Percent volumetric density was analysed in pre-defined categories 0 to 4.5%, ≥4.5 to 7.5%, ≥7.5 to 15.5%, and ≥15.5%, which map to the BI-RADS breast composition categories based on their 4 groupings of: 1-fatty (VDG 1); 2-scattered (VDG 2); 3-heterogenous (VDG 3); and 4-extremely dense (VDG 4) [17]. Age was defined as a subject’s age at the time of the mammogram; only the screening mammogram was included. As no other risk factor information was available from the routinely collected data held by BSWN, the volume of non-dense tissue was used as a proxy measure for BMI in the analyses.

The MLO views were selected for measurement using the combined average for the left and right breast screen image estimates. Descriptive analyses used medians and inter-quartile ranges because of the positively skewed nature of the outcome variables (data available on request). For multivariable analyses, the natural logarithm of the absolute and percentage density values were used. Linear regression of these logged variables was used, and the output was back-transformed and expressed as the ratio of the geometric means. Covariates included in the models were age (as a continuous variable), x-ray system and for absolute density, the natural log of the volume of non- dense tissue.

Statistics were performed using STATA version 11.2.

## Results

Mammographic density measures were available for a total of 4,300 women screened during the pilot study period. These included assessment mammograms for 61 of the women which were subsequently excluded from the final analyses, leaving a total of 4,239. Of these, 3,091 (73.0%) were Pakeha, 716 (7.4%) were Māori, 170 (4.0%) were Pacific and 662 (15.6%) were Asian women.

The median age was highest in Pakeha women at 55 years while Asian women had the lowest median age at 52 years (table 1). The data suggest that total breast volume and thickness differed across ethnic groups being smallest in Asian women and greatest in Pacific followed by Māori women. Māori women the highest abolute density volume, followed by Pacific and Pakeha with Asian women having the lowest absolute density volume. Non-density volume was highest in Pacific and Māori women. Asian women had the highest percent density followed by Pakeha women, while Māori were marginally lower and Pacific women had the lowest percent density. The same patterns of differences were oberved for the two age categories, except that younger Pacific and Pakeha had higher absolute density volumes than Māori women.

**Table 1:** Volumetric mammographic density measures by ethnicity and age.

	Ethnicity							
Variable	Pakehamedian	IQR	Māorimedian	IQR	Pacificmedian	IQR	Asianmedian	IQR
Age (y)	55	49–62	53	48–60	52.5	48–59	52	49–58
breast volume (cm^3^)	923.7	587.4–1358.1	984.3	670.0–1383.1	1106.5	790.4–1447.4	604.2	418.4–893.3
breast thickness (mm)	59.0	47.5–69.0	61.0	51.0–70.3	63.3	54.5–71.5	52.5	44.5–60.5
density volume (absolute) (cm^3^)	49.9	37.8–69.8	53.7	42.8–75.1	51.3	38.5–70.3	45.8	33.5–65.7
non-density volume (cm^3^)	863.7	527.9–1294.7	917.7	620.5–1328.5	1049.6	734.0–1378.6	557.1	377.2–845.2
density percentage (%)	5.6	3.4–9.8	5.5	3.9–8.8	4.7	3.2–7.4	8.1	5.0–12.0
*Age* ≤*50 years*		*N* = 986		*N* = 123		*N* = 69		*N* = 255
breast volume (cm^3^)	854.6	535.3–1337.6	1010.3	642.9–1474.2	1045.4	711.4–1427.8	584.6	422.6–869.2
breast thickness (mm)	58.0	45.5–70.0	61.5	53.5–73.0	64.0	53.5–72.5	52.0	44.5–61.0
density volume (absolute) (cm^3^)	60.1	43.2–82.0	59.1	45.8–82.3	60.2	44.4–84.6	53.5	39.1–77.6
non-density volume (cm^3^)	789.1	469.9–1256.5	910.5	579.8–1401.2	977.3	652.1–1379.0	523.9	374.6–818.1
density percentage (%)	7.9	4.4–12.6	6.3	3.8–11.7	6.3	3.4–9.2	10.2	6.7–13.8
*Age >50 years*		*N* = 2,105		*N* = 193		*N* = 101		*N* = 407
breast volume (cm^3^)	948.3	622.1–1368.1	970.8	694.9–1372.4	1161.8	823.8–1447.4	625.3	410.8–898.5
breast thickness (mm)	59.5	48.5–68.5	61.0	49.5–67	63.0	55.0–70.5	53.0	44.5–60.0
density volume (absolute) (cm^3^)	46.7	36.1–62.1	51.0	41.1–70.2	49.4	37.4–62.2	42.4	31.1–56.1
non-density volume (cm^3^)	895.2	573.7–1315.6	921.8	643.3–1299.7	1111.0	783.0–1376.3	578.1	378.6–848.1
density percentage (%)	5.0	3.2–8.2	5.3	3.9–7.8	4.3	2.9–5.8	6.7	4.5–10.3

Further exploration showed ethnic differences in the distribution of absolute mammographic density both overall and by age categories (table 2). Overall, our results suggest that Māori women were most likely to be in the highest (25%) and least likely to be in the lowest (11%) density quintile in contrast to Asian women who were most likely to be in the lowest and least likely to be in the highest quintiles (28% and 16% respectively). Pakeha and Pacific women were intermediate. These trends were most pronounced in women >50 years. Among younger women, women had denser breasts with Māori, Pacific and Pakeha between 31–32% vs Asian at 26% in the highest quintile, while in the lowest, Māori, Pacific and Pakeha ranged from 9–13% with Asian women at 20%.

**Table 2:** Number (and %) of women in each category of absolute volumetric mammographic density by ethnicity and age.

	Quintile absolute density											
	Q1 (≤34.8%)		Q2 (33.9–44.5%)		Q3 (44.6–55.9%)		Q4 (56.0–75.1%)		Q5 (≥75.2%)		Total	
	*N*	%	*N*	%	*N*	%	*N*	%	*N*	%	*N*	%
*All ages*											4239	
Pakeha	598	19.4	623	20.2	617	19.9	626	20.3	627	20.3	3091	100
Māori	35	11.1	56	17.7	80	25.3	66	20.9	79	25.0	316	100
Pacific	27	15.9	38	22.4	30	17.7	38	22.4	37	21.8	170	100
Asian	187	28.3	131	19.8	121	18.3	118	17.8	105	15.9	662	100
p<0.001												
*Age* ≤*50*											1433	
Pakeha	128	13.0	142	14.4	177	17.9	224	22.7	315	31.9	986	100
Māori	11	8.9	16	13.0	28	22.8	30	24.4	38	30.9	123	100
Pacific	8	11.6	12	17.4	11	15.9	16	23.2	22	31.9	69	100
Asian	50	19.6	39	15.3	45	17.6	55	21.6	66	25.9	255	100
p = 0.287												
*Age >50*											2806	
Pakeha	470	22.3	481	22.8	440	20.9	402	19.1	312	14.8	2105	100
Māori	24	12.4	40	20.7	52	26.9	36	18.6	41	21.2	193	100
Pacific	19	18.8	26	25.7	19	18.8	22	21.8	15	14.8	101	100
Asian	137	33.7	92	22.6	76	18.7	63	15.5	39	9.6	407	100
p<0.001												

Different patterns by ethnicity were also observed in the density percentage categories (table 3) with the data suggesting that Pacific women overall, were most likely (47.7%) to be in the lowest category of percent density and least likely (2.4%) to be in the highest category, with these same patterns of differences also seen in the two age categories. Similarly, Asian women overall were least likely (11.9%) to be in the highest and most likely (19.5%) to be in the lowest percent density categories which was also seen for both the younger and older age categories.

**Table 3:** Number (and %) of women in each category of volumetric percent mammographic density by ethnicity and age.

	Category of density percentage									
	<4.5%		≥4.5 to 7.5%		≥7.5 to 15.5%		≥15.5%		Total	
	*N*	%	*N*	%	*N*	%	*N*	%	*N*	%
*All ages*									4239	
Pakeha	1186	38.4	785	25.4	864	28.0	256	8.3	3091	100
Māori	108	34.2	102	32.3	85	26.9	21	6.7	316	100
Pacific	81	47.7	47	27.7	38	22.4	4	2.4	170	100
Asian	129	19.5	175	26.4	279	42.2	79	11.9	662	100
p<0.001										
*Age* ≤*50*									1433	
Pakeha	259	26.3	219	22.2	363	36.8	145	14.7	986	100
Māori	38	30.9	32	26.0	39	31.7	14	11.4	123	100
Pacific	25	36.2	15	21.7	27	39.1	2	2.9	69	100
Asian	30	11.8	45	17.7	130	51.0	50	19.6	255	100
p<0.001										
*Age >50*									2806	
Pakeha	927	44.0	566	26.9	501	23.8	111	5.3	2105	100
Māori	70	36.3	70	36.3	46	23.8	7	3.6	193	100
Pacific	56	55.5	32	31.7	11	10.9	2	2.0	101	100
Asian	99	24.3	130	31.9	149	36.6	29	7.1	407	100
p<0.001										

The crude associations between ethnicity and absolute volumetric breast density are further shown in Table 4. The higher absolute density in Māori compared to Pakeha women persisted following adjustment for age and x-ray system. Adjusting for non-dense volume, as a proxy for BMI, slightly attenuated the ratio of geometric means. This association was stronger for older compared to younger Māori women. Among Pacific women, there appeared to be little difference in absolute density compared to Pakeha women, although in the fully adjusted model, there was a suggestion that Pacific women were less likely to have higher density. This effect was similar across the two age groups. Asian women, particularly those in the older age group, had lower absolute volumetric breast density compared to Pakeha women, although a substantial part of this effect was due to breast size; after adjustment for non-dense volume the effect was attenuated.

**Table 4:** Unadjusted and adjusted ratio of geometric means for volumetric absolute mammographic density by ethnicity and age.

All women		(95% CI)	*Age* ≤*50*		(95% CI)	*Age >50*		(95% CI)
*Unadjusted*			*Unadjusted*			*Unadjusted*		
Pakeha	1	reference	Pakeha	1	reference	Pakeha	1	reference
Māori	1.09	(1.03–1.15)	Māori	1.03	(0.93–1.13)	Māori	1.10	(1.03–1.17)
Pacific	1.03	(0.96–1.11)	Pacific	1.01	(0.90–1.15)	Pacific	1.01	(0.92–1.10)
Asian	0.89	(0.86–0.93)	Asian	0.90	(0.84–0.97)	Asian	0.86	(0.82–0.91)
*Adjusted for age*			*Adjusted for age*			*Adjusted for age*		
Pakeha	1	reference	Pakeha	1	reference	Pakeha	1	reference
Māori	1.10	(1.01–1.12)	Māori	1.02	(0.93–1.13)	Māori	1.09	(1.02–1.16)
Pacific	1.00	(0.94–1.08)	Pacific	1.01	(0.89–1.15)	Pacific	1.00	(0.92–1.10)
Asian	0.86	(0.83–0.90)	Asian	0.90	(0.84–0.97)	Asian	0.84	(0.80–0.88)
*Adjusted for age and x-ray system*			*Adjusted for age and x-ray system*			*Adjusted for age and x-ray system*		
Pakeha	1	reference	Pakeha	1	reference	Pakeha	1	reference
Māori	1.09	(1.04–1.16)	Māori	1.07	(0.97–1.18)	Māori	1.13	(1.05–1.20)
Pacific	0.99	(0.92–1.07)	Pacific	0.99	(0.88–1.12)	Pacific	0.99	(0.91–1.09)
Asian	0.85	(0.81–0.88)	Asian	0.88	(0.82–0.95)	Asian	0.85	(0.81–0.89)
*Adjusted for age, x-ray system and non-density volume*			*Adjusted for age, x-ray system and non-density volume*			*Adjusted for age, x-ray system and non-density volume*		
Pakeha	1	reference	Pakeha	1	reference	Pakeha	1	reference
Māori	1.06	(1.01–1.12)	Māori	1.02	(0.94–1.12)	Māori	1.10	(1.04–1.17)
Pacific	0.93	(0.87–1.00)	Pacific	0.92	(0.82–1.03)	Pacific	0.94	(0.87–1.02)
Asian	0.95	(0.91–0.99)	Asian	0.98	(0.91–1.04)	Asian	0.96	(0.92–1.01)

A different pattern of associations was seen with volumetric percent density, shown in table 5. Asian women had the greatest risk of high percent breast density of any of the ethnic groups. Adjustment for age had no effect on younger but attenuated the effect in older Asian women, while adjusting for x-ray system strengthened the effect in older and attentuated the effect in younger Asian women. Younger Māori women had a lower percent density than younger Pakeha women, although the effect was partially explained by the different x-ray system used. Conversely, older Māori women had a higher percent breast density, again partially explained by the x-ray system. Pacific women in both age groups had the lowest percentage breast density of any of the population groups which changed only minimally following adjustment. Given that non-dense volume was so highly correlated with overall breast volume, and the latter was used in the calculation of percent breast density, these results were not adjusted for non-dense volume.

**Table 5:** Unadjusted and adjusted ratio of geometric means for volumetric percent mammographic density by ethnicity and age.

All women		(95%CI)	*Age* ≤*50*		(95%CI)	*Age >50*		(95% CI)
*Unadjusted*			*Unadjusted*			*Unadjusted*		
Pakeha	1	reference	Pakeha	1	reference	Pakeha	1	reference
Māori	1.03	(0.95–1.12)	Māori	0.89	(0.78–1.01)	Māori	1.09	(0.99–1.20)
Pacific	0.82	(0.74–0.92)	Pacific	0.78	(0.66–0.92)	Pacific	0.81	(0.71–0.93)
Asian	1.35	(1.27–1.43)	Asian	1.29	(1.17–1.42)	Asian	1.34	(1.25–1.43)
*Adjusted for age*			*Adjusted for age*			*Adjusted for age*		
Pakeha	1	reference	Pakeha	1	reference	Pakeha	1	reference
Māori	1.00	(0.93–1.08)	Māori	0.89	(0.78–1.01)	Māori	1.08	(0.98–1.19)
Pacific	0.80	(0.72–0.89)	Pacific	0.78	(0.66–0.92)	Pacific	0.81	(0.71–0.92)
Asian	1.29	(1.22–1.37)	Asian	1.29	(1.17–1.42)	Asian	1.30	(1.21–1.40)
*Adjusted for age and x-ray system*			*Adjusted for age and x-ray system*			*Adjusted for age and x-ray system*		
Pakeha	1	reference	Pakeha	1	reference	Pakeha	1	reference
Māori	1.00	(0.92–1.08)	Māori	0.93	(0.82–1.07)	Māori	1.06	(0.96–1.16)
Pacific	0.80	(0.72–0.89)	Pacific	0.76	(0.64–0.90)	Pacific	0.83	(0.72–0.94)
Asian	1.30	(1.22–1.37)	Asian	1.26	(1.14–1.39)	Asian	1.36	(1.27–1.46)

## Discussion

These findings have highlighted differential patterns of breast density between ethnic groups in New Zealand. In particular, compared to Pakeha women, Māori women had higher levels of absolute volumetric density and Asian women had lower levels. For percentage breast density, Asian women had higher and Pacific women had lower breast density.

The main limitation of the study is our lack of additional data in order to assess the potential effect of confounding on our results. While imperfect, the estimate of non-dense volume as a proxy for BMI is not unreasonable since body weight is associated with the volume of non–dense tissue in the mammogram [9]. We also acknowledge the fact that, relative to the composition of the NZ population as a whole, our sample included fewer Māori and Pacific women and was over-represented for Asian women. A strength of the study however was that complete information, including mammography data, was available for all women screened during the data collection period.

Our finding of a higher absolute dense volume in Māori compared to Pakeha women remained following adjustment for age, x-ray system and non-dense volume. To date, international literature on differences in breast density by ethnicity is scarce and mixed. Most of the previous work documenting ethnic differences in mammographic density has been conducted in the US [13]–[15], [28]–[31] with one study in the UK also reporting differences by ethnic group [12]. Among the US studies, most have reported lower breast density in African American compared to white American women [13], [28]. One study amongst Alaskan peoples reported Eskimo women to be more likely to have a high breast density compared to Aleut women [32]. In a comparison of seven regional/ethnic groups [14] Hawaiian women were found to have the largest dense breast area and a high area percent breast density, second only to Japanese women in Hawaii [15]. Of note, that latter study is, to our knowledge, the only study of breast density to have been undertaken among Polynesian women, with the current pilot study extending this to now include Māori and other Pacific Island women. Our results for Asian women, who had the lowest absolute density of any of the ethnic groups, is most likely explained by their overall smaller body, and breast, size. In New Zealand, the largest Asian ethnic group is Chinese followed by Indian and Korean [25]. Asian (predominantly of Japanese and Chinese origin) women in US studies have been found to have both lower [28] and higher [13], [29] breast density than white women, with the inconsistent results likely affected by the method used to determine breast density and also confounding by differing mean age at menopause [13].

With regard to percent density, we found a high percent density in Asian and low percent density in Pacific women which is again, most probably due to body fat, although of note, for Māori women, who have an on average higher BMI than Pakeha women [33], we found only a marginally higher percent density. It has been suggested that percent density may not be a good predictor of ethnic differences because it is greatly influenced by breast size and shows marked variation by ethnic group [28]. Recent work on breast density has emphasised the importance of measuring absolute density as well as percentage density in order to overcome the negative association seen with BMI and percentage density - potentially resulting in an underestimation of the risk estimates if only a percent density measure is used [10]. Large variations in measures of women with a high BMI, particularly for absolute density, have been documented [10] which may reflect the fact that women with higher BMI generally have larger breasts.

A further key area of future exploration will be whether the demonstrated differences in breast density can explain ethnic differences in breast cancer risk in New Zealand. Although we were unable to explore this in the current study, our results are generally consistent with documented differences in breast cancer incidence rates seen in this country [2]–[3]. Differences in incidence between Pakeha and Pacific women are explicable in terms of known risk factors [3] but reasons for the increasing incidence among Māori women, are difficult to understand. Screening coverage for the geographic region included in our study is approximately the same as the national average however, the documented lower uptake of screening in Māori compared to non-Māori women [34], in the absence of data to assess relative change in screening rates between these populations, would suggest that screening does not explain the noted higher incidence. Additionally, Māori have a more favourable profile than non-Māori for several breast cancer risk factors including higher parity, younger age at first pregnancy and lower rates of hormone therapy use [35]–[36]. Breast density has also been identified as an important predictor of the accuracy of screening mammography [23], [37]. One implication, if breast density differs by ethnicity, is that there would be differences in breast cancer detection rates by ethnicity, particularly in terms of interval cancer rates. Again, this is an area which could be explored further in future studies in NZ. We were able to take advantage of new technology for assessing breast density, available for use as a research tool by BSWN. Volumetric density measures could potentially be utilised, for example, to assist with optimizing breast screening intervals [38] consistent with recommendations from the International Screening Network who have called for inclusion of data on breast density in screening programmes as a potential means for monitoring their effectiveness for reducing breast cancer mortality [22].

In summary, in these novel analyses of breast density distribution in New Zealand women, we found not only expected age differences in breast density but also differences by ethnicity, with Māori being more likely and Asian women less likely, to have high volumes of dense tissue than Pakeha women. Further work in this area is warranted, in particular, examining the role of breast density and its potential contribution to New Zealand’s well-known, but poorly explained ethnic disparities in breast cancer incidence and outcomes.
